# Upregulation of CD20 Positive B-Cells and B-Cell Aggregates in the Tumor Infiltration Zone is Associated with Better Survival of Patients with Pancreatic Ductal Adenocarcinoma

**DOI:** 10.3390/ijms21051779

**Published:** 2020-03-05

**Authors:** Maximilian Brunner, Katharina Maier, Petra Rümmele, Anne Jacobsen, Susanne Merkel, Alan Benard, Christian Krautz, Stephan Kersting, Robert Grützmann, Georg F. Weber

**Affiliations:** 1 Department of General and Visceral Surgery, Friedrich-Alexander-University, Krankenhausstraße 12, 91054 Erlangen, Germany; Maximilian.Brunner@uk-erlangen.de (M.B.); kmb.maier@gmx.de (K.M.); Anne.Jacobsen@uk-erlangen.de (A.J.); Susanne.Merkel@uk-erlangen.de (S.M.); alan.benard@uk-erlangen.de (A.B.); christian.krautz@uk-erlangen.de (C.K.); Stephan.Kersting@uk-erlangen.de (S.K.); robert.gruetzmann@uk-erlangen.de (R.G.); 2 Department of Pathology, Friedrich-Alexander-University, Krankenhausstraße 8–10, 91054 Erlangen, Germany; petra.ruemmele@uk-erlangen.de

**Keywords:** CD20, B-cells, B-cell aggregates, pancreatic ductal adenocarcinoma, survival

## Abstract

Patients with pancreatic ductal adenocarcinoma (PDAC) normally have a poor long-term prognosis. However, some rare cases of long-term survivors have been reported. The tumor microenvironment, consisting of cellular and stromal components, possibly plays an important role and might influence prognosis. In this context, the role of tumor-infiltrating B-cells and its impact on the survival in patients with PDAC remains controversial. We therefore aimed to assess the prognostic value of CD20-positive B-cells and CD20-positive B-cell aggregates as well as CD138, IgM, Pax5, and Ki67 on the survival of patients with PDAC using immunohistochemistry of FFPE pancreatectomy tissue sections from patients that underwent primary surgery for pT3- and R0-pancreatic adenocarcinoma between 1995 and 2016. Patients with PDAC were matched and grouped in 16 long-term-survivors (LTS, median overall survival (OS): 96 months [range: 61–177 months]) and 16 short-term-survivors (STS, median OS: 16 months [range: 7–32 months]). CD20-positive B-cells and B-cell aggregates in the tumor infiltration zone were significantly upregulated in the LTS-group compared to the STS-group (*p* = 0.0499 respectively *p* = 0.0432). Regarding the entire patient cohort (*n* = 32) CD20 positive B-cell aggregates in the tumor infiltration zone were an independent prognostic marker for overall survival in multivariate analysis (HR 9.2, CI 1.6–51.4, *p* = 0.012). These results underline the importance of tumor-associated B-cells for prognosis of patients with PDAC. The detailed role of B cells in the pathomechanism of PDAC should be further investigated for predicting outcome, identifying appropriate treatment regimens, and developing novel therapeutic options.

## 1. Introduction

Pancreatic ductal adenocarcinoma (PDAC) is associated with a poor prognosis, accounting for the seventh leading cause of cancer-related mortality worldwide [1]. In 2018, PDAC was diagnosed in about 458,000 people worldwide and more than 432,000 died of this disease in the same year [2]. The 5- and 10-year survival rates are low, ranging in Germany from 4% to 17% and 2% to 12%, respectively [3]. Although progress has been made in multimodal treatment approaches, the mortality rate of PDAC is still increasing throughout the years. Surgery is considered the only potential curative treatment for PDAC but is reserved for the minority of patients with non-metastatic and locally resectable tumors. Most patients with PDAC remain asymptomatic until the disease develops to an advanced inoperable stage, leading to its disappointing prognosis [4].

The determination of prognostic factors in patients with PDAC is essential for predicting the outcome and for identifying appropriate treatment strategies. Known clinical prognostic parameters include age, tumor stage, lymph node status, grading, perineural invasion, and for resected patients’ resection status, adjuvant chemotherapy and hospital volume [5,6,7].

Moreover, it has long been recognized that the tumor microenvironment and an involvement of the immune system play a distinctive role in the biological behavior of cancer [8]. PDACs are characterized by an immunosuppressive microenvironment due to the dysfunction of the immune system, which is a result of the involvement of multiple types of immune cells, including cancer-associated fibroblasts, regulatory T cells, myeloid-derived suppressor cells, tumor-associated macrophages, and tumor-infiltrating lymphocytes [9]. Until now, most studies have largely focused on the T cell compartment. The function of tumor-infiltrating B-cells, however, and its impact on survival in patients with PDAC, remains unclear [10,11].

The aim of the present study was to examine the prognostic role of B-cells and B-cell aggregates on the survival of patients with PDAC using CD20—a transmembrane phosphoprotein that is expressed on B-lymphocytes in different stages of development [12]. Additionally, the following B-cell associated markers were investigated: CD138—a transmembrane receptor, which participates in cell proliferation, cell migration, and cell-matrix interactions and is also known as syndecan-1 [13]; IgM—an immunoglobulin that occurs on the surface of B-cells as well as freely circulating in the blood [14]; Pax 5—a nuclear transcription factor, which is required for B cell development [15]; and Ki67—a nuclear protein expressed by cells in the proliferative phase [16].

## 2. Materials and Methods

### 2.1. Patients

We analyzed patients with previously untreated PDAC, who underwent primary surgery at the university hospital Erlangen, Germany, during the period between 1995 and 2016. Patients included in this analysis had to meet following additional criteria: Complete macroscopic and microscopic surgical resection (R0), pT3-category in histopathological examination, no in-hospital-mortality, and survival of a minimum of one month.

Patients’ clinical and pathological data were obtained from the Erlangen Cancer Registry of the Department of Surgery. The detailed documentation allowed a classification of pathology and staging of all patients according to the eighth edition of the tumor–node–metastasis (TNM) classification system [17].

**Figure 1:**
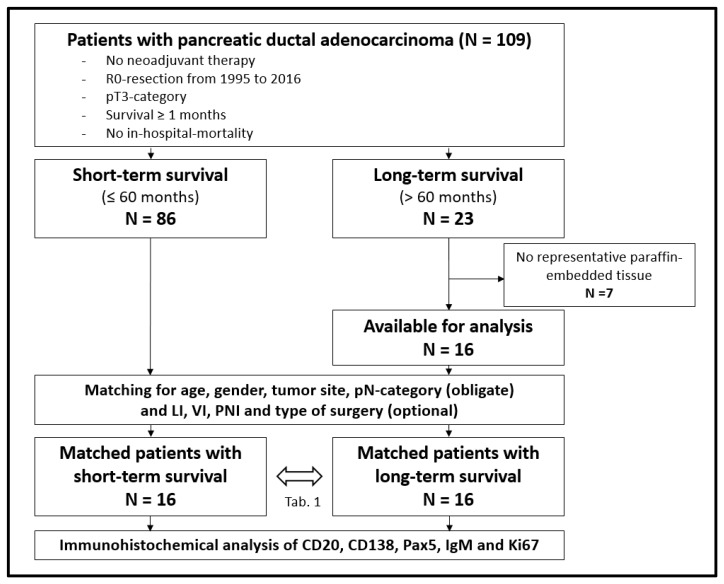
Flow chart for patient inclusion; LI = lymphatic invasion, VI = vascular invasion, PNI = perineural invasion.

A total of 109 patients met the inclusion criteria. Eighty-six of the 109 patients had a survival of no longer than 60 months (short-term survival (STS)), while 23 patients showed a survival over 60 months (long-term survival (LTS)). After exclusion of seven patients with no representative formalin-fixed paraffin-embedded surgical samples in the LTS-groups, 16 LTS-patients were matched with the STS-group. Obligate matching criteria were age (difference of up to 9 years), gender, tumor site, and histopathological *p*N-category. In addition, matching of lymphatic invasion, vascular invasion, perineural invasion, grading, and type of surgery was carried out as far as possible. There were at least 16 matched patients in each group for immunohistochemical analysis (Figure 1).

### 2.2. Immunohistochemical Staining and Scoring

Formalin-fixed paraffin-embedded tissue blocks of the 32 patients were anonymously reviewed by an experienced pathologist and the most representative block of each patient with the most inflammation in the tumor infiltration zone was selected for immunohistochemical staining.

Sections (1 µm) of the paraffin-embedded tissue blocks were cut with Leica microtom and mounted on slides. Slides were stained with hematoxylin and immunohistochemically with the primary antibodies CD20 (Dako, Ely, UK), CD138 (Zytomed Systems, Berlin, Germany), IgM (Dako, Ely, UK), Pax5 (BD Biosciences, Barking, UK), and Ki67 (Dako, Ely, UK) using the Benchmark Ultra system (Roche, Mannheim, Germany). Subsequently, slides were stained with the chromogen DAB (Roche, Mannheim, Germany) followed by a hemtoxylin and bluing reagent counterstain (Roche, Mannheim, Germany). Staining of a positive and a negative tissue control was done.

**Figure 2:**
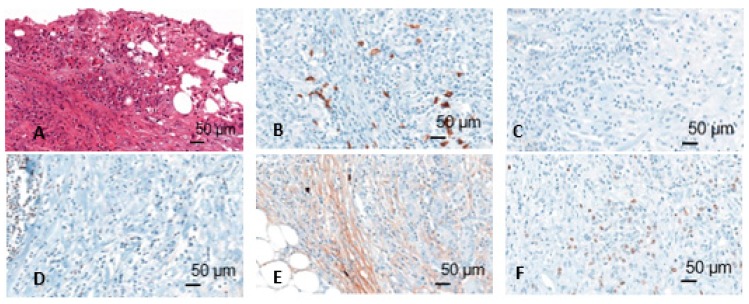
Different immunohistochemical stainings (HE (**A**), CD20 (**B**), CD138 (**C**), Pax5 (**D**), IgM (**E**), Mib (**F**)); figures from the dissertation of KM.

Immunohistochemically stained slides were scanned by Panoramic 250 Flash II scanner (3D Histech, Budapest, Hungary) at 40× magnification. The scans were blindly analyzed using CaseViewer software. First, the tumor infiltration zone was determined on the basis of the HE staining. After evaluation of the positive and negative tissue controls, the assessment of expression of CD20, CD138, Pax5, IgM, and Ki67 was performed (Figure 2). For CD20, CD138, Pax5, and IgM 10 counting areas were defined at 10× magnification and the positive cells in these areas were counted at 20× magnification. For Ki67, 500 cells were counted, and a quotient of positive and negative cells was formed. Cells were assessed in two compartments: Tumor and tumor infiltration zone. Moreover, CD20 positive lymphocytes aggregates in the tumor infiltration zone were measured.

### 2.3. Statistical Analysis

Data analysis was performed with SPSS software (SPSS Inc., Chicago, IL, USA). Comparisons of metric and ordinal data were calculated with the Student *t*-test or Mann Whitney U test. The Chi-square test was used for categorical data. Statistical significance was set at *p* < 0.05. Overall survival was calculated from the date of surgery to the day of death or last follow-up. The Kaplan–Meier method was used to plot the survival curves and Log-Rang test to compare influencing factors in univariate analysis. For correlation analysis Spearman coefficient was determined meaning 0.0 to ≤0.2 no correlation, >0.2 to ≤0.5 moderate correlation, and >0.5 high correlation. The results of the immunohistochemical markers (CD20, CD20-positive aggregates in the tumor compartment, CD20-positive aggregates in the tumor infiltration zone, CD138, IgM, Pax5, and Mib) were assessed for outliers using GraphPad QuickCalcs. Three outliers for CD20 and Pax5 and one outlier for IgM and CD138 were excluded from analysis due to insufficient staining. Dichotomized labelling (low vs. high expression) was based on the median value of immune marker expression. Multivariate analysis was performed using Cox regression model with backward elimination, including a log-likelihood adjustment. Backward elimination was applied with inclusion of all immunhistochemical parameters from the starting model. Variables were included if the *p*-values were less than 0.05 and were removed if the *p*-values were greater than 0.10.

## 3. Results

### 3.1. Clinicopathological Characteristics

The study cohort includes 32 patients (mean age 64.7 years [range 47–78], 69% female) with mainly in the head localized PDACs grouped into 16 short- (STS) and 16 long-term-survivors (LTS). Next to the obligate matching parameters type of surgery, grading, lymphatic, vascular and perineural invasion, as well as frequency of adjuvant chemotherapy, CA19-9 and CEA did not differ between the two groups. Adjuvant chemotherapy was performed in 34% of patients using gemcitabine alone in 45% of adjuvant treated patients and a gemcitabine-based combination therapy in 45% of adjuvant-treated patients (Table 1).

### 3.2. Immune Marker and Lymphoid Aggregates

**Figure 3:**
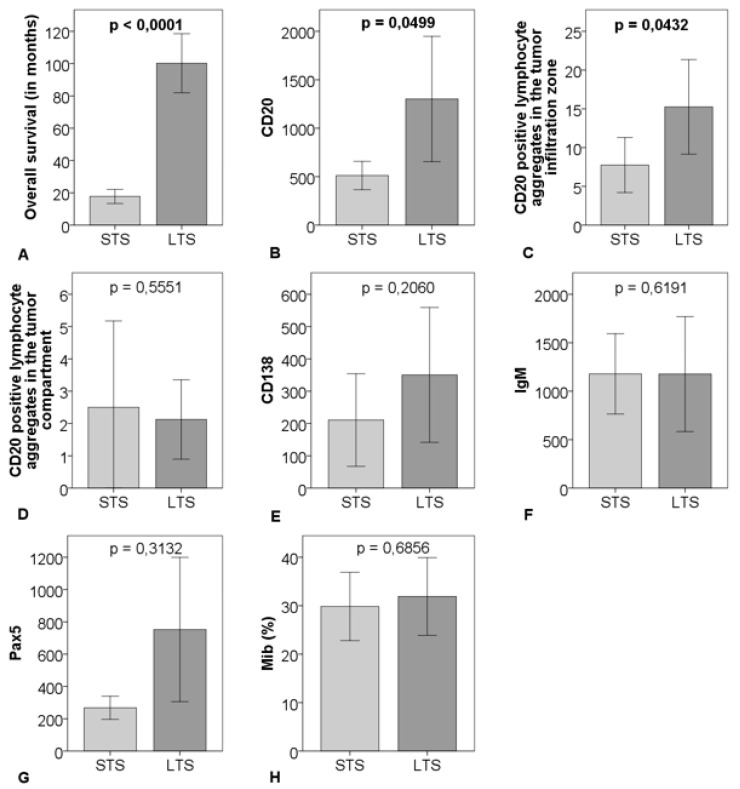
Comparison of survival (**A**), cell count of CD20 (**B**), CD20-positive B-cell aggregates in the tumor infiltration zone (**C**), and in the tumor compartment (**D**), CD138 (**E**), IgM (**F**), Pax5 (**G**), percentage of Mib (**H**) between 16 matched short-term survivors (STS) and 16 matched long-term survivors (LTS); outliner excluded > CD20: *n* = 29, CD138: *n* = 31, IgM: *n* = 31, Pax5: *n* = 29; the graphs show mean and 95% CI including *p*-value.

**Figure 4:**
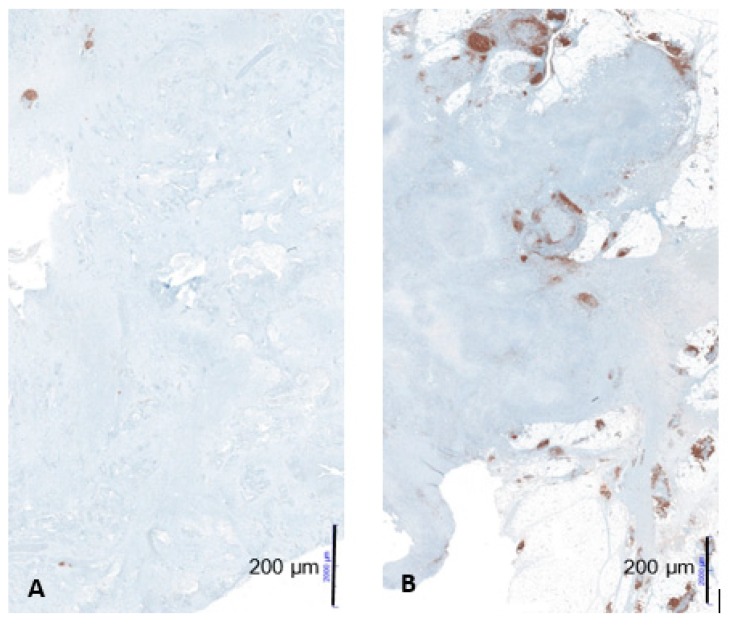
Example of low CD20-positive B-cell aggregates in a patient with short-term survival (**A**) and high CD20-positive B-cell aggregates in a patient with long-term survival (**B**) at 10× magnification; figures from the dissertation of KM.

CD20-positve B-cells and CD20-positive B-cell aggregates in the tumor infiltration zone were significantly upregulated in the LTS-group compared to the STS-group (*p* = 0.0499 respectively *p* = 0.0432). There were no significant differences regarding CD20-positive B-cell aggregates in the tumor compartment, Pax5, CD138, Mib, and IgM comparing the two groups (Figure 3 and Figure 4).

In the LTS-group, CD20 showed a high correlation with CD20-positive B-cell aggregates in the tumor infiltration zone, Pax 5, CD138, Mib, and IgM (spearman rho: 0.6124, 0.9029, 0.5857, 0.6357, and 0.5956, respectively). In the STS-group, CD20 correlated highly with CD20-positive B-cell aggregates in the tumor infiltration zone, Pax 5, and CD138 (spearmen rho: 0.5709, 0.8542, and 0.5209, respectively). Moderate and no correlation existed between CD20 and Mib and IgM, respectively, in the STS-group (spearman rho: 0.4813 and 0.1298, respectively).

### 3.3. Correlation of Immune Marker Expression with Clinicopathological Characteristics

Regarding the impact of clinicopathological characteristics on the expression of the investigated markers (CD20, CD20-positive B-cell aggregates in the tumor compartment and in the tumor infiltration zone, CD138, IgM, Pax5, and Mib), there was a significant association between high CD20-expression and an age ≥65 years (*p* = 0.025). High IgM-expression was more often found in the pancreatic head and therefore associated with a higher rate of pancreatic head resections (*p* = 0.018 and *p* = 0.024, respectively) (Table 2).

### 3.4. Multivariate Analysis

Regarding the entire patient cohort (*n* = 32), high expression of CD20-positive B-cell aggregates in the tumor infiltration zone was an independent prognostic marker for overall survival in multivariate analysis (HR 9.2, CI 1.6–51.4, *p* = 0.012). The other investigated immune markers showed no significant impact on overall survival in multivariate analysis (Table 3).

## 4. Discussion

The tumor microenvironment is known to be an important parameter influencing the biological behavior of carcinomas and therefore prognosis of cancer patients. Several studies revealed especially tumor-associated lymphocytes (TALs) to be associated with good prognosis in pancreatic cancer as well as in different solid tumors [8,18,19,20,21,22] but focused largely on the T cell compartment. However, the role of B-cells and its impact on survival in patients with PDAC remains controversial [10,11].

CD20 is a 33–37 kDa transmembrane phosphoprotein, which is expressed on B-lymphocyte precursors and mature B-lymphocytes. CD20 positive B-cell infiltration has been associated with improved patient survival as well as increased immunotherapy response in various cancers [23,24]. Until now, there were only two studies investigating the association of CD20-positive B-cells with survival in patients with pancreatic carcinomas. Tewari et al. examined 81 patients with pancreatic ductal carcinomas and showed that high CD20-positive lymphocyte levels were associated with an improved survival [10]. In contrast, an investigation of pancreatectomy tissue sections of 141 primary resected pancreatic ductal adenocarcinomas by Diana et al. revealed no correlation of CD20 and prognostic outcome [11].

Our study is the first analysis comparing matched groups. In our cohort, CD20-positive B-cells and CD20-positive B-cell aggregates in the tumor infiltration zone were significantly upregulated in the LTS-group compared to the STS-group indicating a positive association of B-cells with prognosis. Moreover CD20-positive B-cell aggregates in the tumor infiltration zone were proved to be an independent prognostic marker for overall survival in multivariate analysis (HR 9.2, CI 1.6–51.4, *p* = 0.012).

However, the specific role of B-cells in the pathophysiological mechanism of PDAC remains unclear. The investigated B-cell associated markers (CD138, PAX 5, MiB, IgM) showed no specific pattern in our study and were therefore unable to indicate a potential pathway.

B-cells are known to constitute effector cells and to promote tumor-specific activation of cytotoxic T-cells via antigen presentation [25]: B-cells can internalize antigen that binds to their B-cell receptor and present it to helper T-cells. Unlike T-cells, B-cells can recognize soluble antigen for which their B-cell receptor is specific. Whereas antigen-presenting cells such as dendritic cells and macrophages ingest and present antigens nonspecifically, a B-cell generally presents only an antigen that it specifically recognizes. Naive helper T cells are then activated by binding to a foreign peptide bound to class II MHC proteins on the surface of a dendritic cell. Once activated, the effector helper T cell can then activate a B-cell that specifically displays the same complex of foreign peptide and class II MHC protein on its surface. The display of antigen on the B-cell surface reflects the selectivity with which it takes up foreign proteins from the extracellular fluid. These foreign proteins are selected by the antigen receptors on the surface of the B-cell and are ingested by receptor-mediated endocytosis. They are then degraded and recycled to the cell surface in the form of peptides bound to class II MHC proteins. Thus, the helper T-cell activates those B-cells with receptors that specifically recognize the antigen that initially activated the T-cell, although the T- and B-cells usually recognize distinct antigenic determinants on the antigen. In secondary antibody responses, memory B-cells themselves can act as antigen-presenting cells and activate helper T-cells, as well as being the subsequent targets of the effector helper T-cells. The mutually reinforcing actions of helper T-cells and B-cells lead to an immune response that is both intense and highly specific. Thus, it is reasonable to assume that interaction between various immune cells such as CD20-positive B-cells with T-cells represent an active immune response leading to less tumor immune evasion resulting in a better prognosis in patients with PDAC.

In contrast, there are preclinical studies reporting a protumorigenic role of B-cell subtypes [26,27,28]. Another interesting hypothesis explaining the B-cell-upregulation could be that some of the B-cells could even be malignant, as a recent study showed that in patient-derived xenograft mice models pancreatic cancer tumors can grow as a lymphocytic tumor containing CD20-expressing B-cells [29].

This suggests that the role of B-cells in pancreatic tumorgenesis seems to be more complex and not fully understood. However, the accumulation of B-cells into lymphoid aggregates close to T- and other cells seems to play a decisive prognostic role, as our investigation confirms, although the reasons for this are also not known [11,30].

In our cohort, age ≥65 years was significantly associated with a high CD20 expression. As advanced age is normally associated with a poorer prognosis, it is unclear to what extent age plays a role in CD20 expression. However, high CD20 expression as well as high expression of CD20-positive B-cell aggregates in the tumor infiltration zone were not associated with any other prognosis influencing clinicopathological characteristics like lymph node status (N) or grading (G) indicating a potentially underlying pathway. A possible interesting aspect could also be an impact of B-cells on the efficacy of adjuvant chemotherapy. Unfortunately, an investigation of this was not possible due to the small number of cases and the high heterogeneity of chemotherapeutics.

This study has some limitations. First, the small sample size limited the statistical power and the retrospective design of our study may have incurred some bias. Second the assessment and counting of immune markers is always somewhat arbitrary; therefore, a digital method might improve the reproducibility. Third, the examination of the exact pathomechanism was limited because, due to the retrospective design of the study, there were no blood samples or fresh tissue samples available for blood analysis or mass cytometric analysis, which could have provided even more findings regarding the pathomechanism of B-cells in pancreatic cancer.

## 5. Conclusions

This study is the first to report an association of CD20-positive B-cells with the long-term prognosis of patients with pancreatic ductal adenocarcinomas in an accurately matched cohort. As new promising immune therapies presently arise, the interaction and role of CD20-positive B-cells in the development of pancreatic adenocarcinoma should be further investigated for predicting outcome and develop novel therapy options.

## Figures and Tables

**Table 1:** Clinicopathological characteristics of matched short-term survival patients (STS, *n* = 16) and long-term survival patients (LTS, *n* = 16); data presented as *n* (%); * obligate matching; + 3× Gemcitabine, 2× Gemcitabine + 5 FU, 1× Gemcitabine + Cisplatin + Vinorelbin + Paclitaxel, 1× unknown; # 2× Gemcitabine, 1× Gemcitabine + 5 FU, 1× Gemcitabine + Erlotinib.

		STS	LTS	*p*-Value
Number		16	16	
Mean Age (in years [range])		65 (47–78)	64 (48–78)	0.824 *
Gender	Female	11 (69)	11 (69)	1.000 *
	Male	5 (31)	5 (31)	
Tumor site	Head	13 (81)	13 (81)	1.000 *
	Head and tail	2 (13)	2 (13)	
	Tail	1 (6)	1 (6)	
pN-category	*p*N0	7 (44)	7 (44)	1.000 *
	*p*N +	9 (56)	9 (56)	
Type of surgery	Whipples	12 (75)	12 (75)	0.839
	Pylorus preserving	1 (6)	2 (13)	
	Left pancreatectomy	3 (19)	2 (13)	
Grading	G2	6 (38)	6 (38)	1.000
	G3	10 (63)	10 (63)	
Lymphatic invasion	No	9 (56)	12 (75)	0.458
	Yes	7 (44)	4 (25)	
Vascular invasion	No	15 (94)	14 (88)	1.000
	Yes	1 (6)	2 (13)	
Perineural invasion	No	4 (25)	6 (38)	0.704
	Yes	12 (75)	10 (63)	
Adjuvant chemotherapy	Yes	4 ^#^ (25)	7 ^+^ (44)	0.438
	No	12 (75)	9 (56)	
CA19-9	<40 IE/mL	5 (31)	5 (31)	1.000
	≥40 IE/mL	5 (31)	5 (31)	
	Unknown	6 (38)	6 (38)	
CEA	<5 ng/L	7 (44)	9 (56)	0.311
	≥5 ng/L	4 (25)	1 (6)	
	Unknown	1 (31)	6 (38)	

Median overall survival (OS) was 96 months [range: 61–177 months] in the LTS-group compared to 16 months [range: 7–32 months] in the STS-group.

**Table 2:** Impact of clinicopathological characteristics on CD20, CD20-aggregates, CD138, IgM, Pax5, and Mib expression; data presented as *n* (%); * outliner excluded > CD20: *n* = 29, CD138: *n* =31, IgM: *n* =31, Pax5: *n* =29; bold *p*-values are significant (<0.05); ** data incomplete.

		CD20 *		CD20-agg. TC			CD20-agg. IZ			CD138 *			IgM *			Pax5 *			Mib		
	Low	High	*p*	Low	High	*p*	Low	High	*p*	Low	High	*p*	Low	High	*p*	Low	High	*p*	Low	High	*p*
**Age**																					
<65 years	12 (80)	5 (36)	0.025	11 (58)	7 (54)	1.000	11 (69)	7 (44)	0.285	10 (63)	8 (53)	0.722	10 (63)	8 (53)	0.722	10 (67)	7 (50)	0.462	9 (56)	9 (56)	1000
≥65 years	3 (20)	9 (64)		8 (42)	6 (46)		5 (31)	9 (56)		6 (38)	7 (47)		6 (38)	7 (47)		5 (33)	7 (50)		7 (44)	7 (44)	
**Gender**																					
Female	9 (60)	12 (86)	0.215	13 (68)	9 (69)	1.000	12 (75)	10 (63)	0.704	12 (75)	10 (67)	0.704	9 (56)	13 (87)	0.113	10 (67)	11 (79)	0.682	10 (63)	12 (75)	0.704
Male	6 (40)	2 (14)		6 (32)	4 (31)		4 (25)	6 (38)		4 (25)	5 (33)		7 (44)	2 (13)		5 (33)	3 (21)		6 (38)	4 (25)	
**Tumor Site**																					
Head	11 (73)	13 (93)	0.100	14 (74)	12 (92)	0.505	15 (94)	11 (69)	0.204	13 (81)	12 (80)	0.342	10 (63)	15 (100)	0.018	12 (80)	12 (86)	0.598	13 (81)	13 (81)	0.342
Head and tail	4 (27)	0 (0)		3 (16)	1 (8)		1 (6)	3 (19)		3 (19)	1 (7)		4 (25)	0 (0)		3 (20)	1 (7)		3 819)	1 (6)	
Tail	0 (0)	1 (7)		2 (11)	0 (0)		0 (0)	2 (13)		0 (0)	2 (13)		2 (13)	0 (0)		0 (0)	1 (7)		0 (0)	2 (13)	
***p*N**																					
*p*N0	7 (47)	6 (43)	1.000	10 (52)	4 (31)	0.289	7 (44)	7 (44)	1.000	8 (50)	5 (33)	0.473	8 (50)	5 (33)	0.473	7 (47)	6 (43)	1.000	8 (50)	6 (38)	0.722
*p*N+	8 (53)	8 (57)		9 (47)	9 (69)		9 (56)	9 (56)		8 (50)	10 (67)		8 (50)	10 (67)		8 (53)	8 (57)		8 (50)	10 (63)	
**Type of Surgery**																					
Whipples	11 (73)	11 (79)	0.686	14 (74)	10 (77)	0.497	14 (88)	10 (63)	0.356	13 (81)	10 (67)	0.595	9 (56)	14 (93)	0.040	11 (73)	11 (79)	0.686	13 (81)	11 (69)	0.730
Pylorus preserving	1 (7)	2 (14)		1 (5)	2 (15)		1 (6)	2 (13)		1 (6)	2 (13)		2 (13)	1 (7)		1 (7)	2 (14)		1 (6)	2 (13)	
Left pancreatectomy	3 (20)	1 (7)		4 (21)	1 (8)		1 (6)	4 (25)		2 (13)	3 (20)		5 (31)	0 (0)		3 (20)	1 (7)		2 (13)	3 (19)	
**Grading**																					
G2	5 (33)	6 (43)	0.710	7 (37)	5 (39)	1.000	6 (38)	6 (38)	1.000	5 (31)	7 (47)	0.473	5 (31)	7 (47)	0.473	5 (33)	6 (43)	0.710	6 (38)	6 (38)	1.000
G3	10 (67)	8 (57)		12 (63)	8 (62)		10 (63)	10 (63)		11 (69)	8 (53)		11 (69)	8 (53)		10 (67)	8 (57)		10 (63)	10 (63)	
**Lymphatic Invasion**																					
No	11 (73)	10 (71)	1.000	13 (68)	8 (62)	0.721	11 (69)	10 (63)	1.000	11 (69)	9 (60)	0.716	10 (63)	11 (73)	0.704	10 (67)	11 (79)	0.682	11 (69)	10 (63)	1.000
Yes	4 (27)	4 (29)		6 (32)	5 (39)		5 (31)	6 (38)		5 (31)	6 (40)		6 (38)	4 (27)		5 (33)	3 (21)		5 (31)	6 (38)	
**Vascular Invasion**																					
No	13 (87)	13 (93)	1.000	17 (90)	12 (92)	1.000	15 (94)	14 (88)	1.000	14 (88)	14 (93)	1.000	15 (94)	13 (87)	0.600	13 (87)	13 (93)	1.000	14 (88)	15 (94)	1.000
Yes	2 (13)	1 (7)		2 (11)	1 (8)		1 (6)	2 (13)		2 (13)	1 (7)		1 (6)	2 (13)		2 (13)	1 (7)		2 (13)	1 (6)	
**Perineural Invasion**																					
No	5 (33)	4 (29)	1.000	6 (32)	4 (31)	1.000	4 (25)	6 (38)	0.704	4 (25)	6 (40)	0.458	5 (31)	4 (27)	1.000	4 (27)	5 (36)	0.700	3 (19)	7 (44)	0.252
Yes	10 (67)	10 (71)		13 (68)	9 (69)		12 (75)	10 (63)		12 (75)	9 (60)		11 (69)	11 (73)		11 (73)	9 (64)		13 (81)	9 (56)	
**Adjuvant Chemotherapy**																					
Yes	5 (33)	6 (43)	0.710	6 (32)	5 (39)	0.721	7 (44)	4 (25)	0.458	7 (44)	4 (27)	0.458	5 (31)	6 (40)	0.716	6 (40)	5 (36)	1.000	5 (31)	6 (38)	1.000
No	10 (67)	8 (57)		13 (68)	8 (62)		9 (56)	12 (75)		9 (56)	11 (73)		11 (69)	9 (60)		9 (60)	9 (64)		11 (69)	10 (63)	
CA19-9 **																					
<40 IE/mL	5 (46)	5 (63)	0.650	7 (58)	3 (38)	0.650	7 (58)	3 (38)	0.650	8 (73)	2 (25)	0.070	4 (33)	6 (75)	0.170	6 (50)	4 (57)	1.000	7 (64)	3 (33)	0.370
≥40 IE/mL	6 (55)	3 (38)		5 (42)	5 (63)		5 (42)	5 (63)		3 (27)	6 (75)		8 (67)	2 (25)		6 (50)	3 (43)		4 (36)	6 (67)	
CEA **																					
<5 ng/L	9 (82)	6 (67)	0.617	10 (77)	6 (75)	1.000	7 (64)	9 (90)	0.311	7 (64)	9 (90)	0.311	9 (75)	7 (78)	1.000	10 (83)	5 (63)	0.603	9 (82)	7 (70)	0.635
≥5 ng/L	2 (18)	3 (33)		3 (23)	2 (25)		4 (36)	1 (10)		4 (36)	1 (10)		3 (25)	2 (22)		2 (17)	3 (38)		2 (18)	3 (30)	

**Table 3:** Multivariate analysis of the impact of different immune markers on overall survival in the entire patient cohort (*n* = 32); * outliner excluded > CD20: *n* = 29, CD138: *n* = 31, IgM: *n* = 31, Pax5: *n* = 29; bold values are significant (*p* < 0.05).

	Univariate	Multivariate		
		HR	95% CI	*p*-Value
CD20 * (Low vs. high)	0.462	-	-	0.376
CD20-aggregates in the tumor compartment (Low vs. high)	0.473	-	-	0.337
CD20-aggregates in the tumor infiltration zone (Low vs. high)	**0.016**	**9.2**	**1.6–51.4**	**0.012**
CD138 * (Low vs. high)	0.724	-	-	0.337
IgM * (Low vs. high)	0.289	-	-	0.459
Pax5 * (Low vs. high)	0.462	-	-	0.648
Mib (Low vs. high)	0.724	-	-	0.189

## Abbreviations

PDAC	Pancreatic ductal adenocarcinoma
LTS	Long-term survivors
STS	Short-term survivors
OS	Overall survival
HR	Hazard ratio
CI	Confidence interval
TNM	Tumor-node-metastasis
CA19-9	Carbohydrate antigen 19-9
CEA	Carcinoembryonic antigen
TALs	Tumor-associated lymphocytes
kDa	Kilodalton
MHC	Major histocompatibility complex
LI	Lymphatic invasion
VI	Vascular invasion
PI	Perineural invasion
FU	Fluoruracil
